# A gain of function paradox: Targeted therapy for glioblastoma associated with abnormal NHE9 expression

**DOI:** 10.1111/jcmm.14665

**Published:** 2019-09-18

**Authors:** Ashley E. Pall, Lena Juratli, Dhyana Guntur, Krisanu Bandyopadhyay, Kalyan C. Kondapalli

**Affiliations:** ^1^ Department of Natural Sciences University of Michigan‐Dearborn Dearborn MI USA

**Keywords:** blood‐brain barrier, endosome, glioblastoma, gold nanoparticles, neurological disease, NHE9, pH, photothermal therapy, SLC9A9, sodium‐proton exchanger

## Abstract

Glioblastoma (GBM) is the most frequent and inevitably lethal primary brain cancer in adults. It is recognized that the overexpression of the endosomal Na^+^/H^+^ exchanger NHE9 is a potent driver of GBM progression. Patients with NHE9 overexpression have a threefold lower median survival relative to GBM patients with normal NHE9 expression, using available treatment options. New treatment strategies tailored for this GBM subset are much needed. According to the prevailing model, NHE9 overexpression leads to an increase in plasma membrane density of epidermal growth factor receptors (EGFRs) which consequently enhances GBM cell proliferation and migration. However, this increase is not specific to EGFRs. In fact, the hallmark of NHE9 overexpression is a pan‐specific increase in plasma membrane receptors. Paradoxically, we report that this gain of function in NHE9 can be exploited to effectively target GBM cells for destruction. When exposed to gold nanoparticles, NHE9 overexpressing GBM cells accumulated drastically high amounts of gold via receptor‐mediated endocytosis, relative to control. Irradiation of these cells with near‐infrared light led to apoptotic tumour cell death. A major limitation for delivering therapeutics to GBM cells is the blood‐brain barrier (BBB). Here, we demonstrate that macrophages loaded with gold nanoparticles can cross the BBB, deliver the gold nanoparticles and effect the demise of GBM cells. In combination with receptor tyrosine kinase inhibition, we show this approach holds great promise for a new GBM‐targeted therapy.

## 1 INTRODUCTION

Glioblastoma (GBM) is the most common and lethal primary brain tumour.^1^, ^2^ GBM is classified as Grade IV glioma by the World Health Organization, and patients have a median survival of approximately 14.6 months post‐diagnosis.^3^ Recently, tumour classification is shifting towards utilizing gene indices and molecular correlates. This allows for more precise treatments tailored to patient‐specific tumour subtypes. Combination of therapeutics precisely designed to target these individual subsets of GBM are increasingly becoming the basis of current day anti‐GBM therapies. Mining of databases comprising of genome and proteome changes in GBM patients led to discovery of a subset of GBM associated with NHE9 overexpression.^4^ Notably, tumours in this subset are non‐responsive to radiation and chemotherapy. NHE9 up‐regulation correlated with significantly shorter disease‐free periods post‐tumour resection and decreased patient survival.^4^

NHE9 is a sodium (potassium) proton antiporter encoded by the gene SLC9A9. NHE9 is localized mostly to early and recycling endosomes in glia, where it transports the protons out of the endosomal lumen in exchange for sodium or potassium ions, alkalizing the endosomal lumen in this process.^5^, ^6^ NHE9 is one of the top 12% of overexpressed genes in GBM.^4^ Mechanistic interrogation of patient‐derived glioblastomas indicated NHE9 overexpression led to an  increased number of epidermal growth factor receptors (EGFRs) on the cell surface.^4^ In normal glia, EGFRs are endocytosed and routed for lysosomal degradation, after triggering downstream signalling cascades for cell growth and proliferation.^7^ However, NHE9 overexpression limits luminal acidification to route endocytosed EGFRs to the plasma membrane instead of the lysosomes.^4^ Thus, NHE9 up‐regulation exerts post‐translational control over EGFR turnover, resulting in the persistence of oncogenic signalling pathways that drive tumour growth.^4^ We recently identified down‐regulation of microRNA‐135a as a potential mechanism underlying the high NHE9 expression observed in the GBM subset.^8^

Clinical trials using drugs targeting EGFR kinases have not had very successful outcomes due to various factors including adaptation to dose‐limiting drug toxicity.^9^ NHE9 overexpression in glioblastoma cells was shown to greatly decrease the efficacy of erlotinib, a known FDA‐approved tyrosine kinase inhibitor of EGFR.^4^ The goal of this study was to integrate the knowledge gained from our previous investigations into NHE9 function, to significantly improve the efficacy in the treatment of the subset of GBM associated with NHE9 overexpression (henceforth referred to as GBM9+). The effects of endosomal alkalization due to NHE9 up‐regulation in tumour cells are not restricted to EGFRs. Endosomal NHE9 activity exerts a profound pan‐specific effect on membrane persistence of multiple receptors and transporters, and an overall elevation in endocytic uptake.^4^, ^5^, ^10^ Here, we describe a new therapeutic approach that utilizes this gained function by GBM9+ tumour cells to effectively target them for destruction. Gold nanoparticle‐enabled photothermal therapy (NEPTT) is gaining much interest as a powerful strategy for cancer treatment.^11^ Gold nanoparticles (GNPs) usually consist of a dielectric silica core and a thin gold shell.^12^ GNPs have been shown to accumulate in tumour cells mostly via endocytic pathways.^13^ When irradiated with near‐infrared (NIR) light, GNPs convert the absorbed light into heat, which is detrimental to the cells accumulating these particles.^13^ NIR has the advantage of penetrating deeply through healthy tissue without affecting it.^12^, ^13^

In this study, we demonstrate that NHE9 overexpression results in strong elevation of GNP accumulation in tumour cells and consequently increased susceptibility to NEPTT treatment. In combination with NEPTT, erlotinib can be used at a much lower dosage in GBM9+ tumours, greatly increasing the efficacy of the drug. An important limiting factor for brain cancer therapy is the blood‐brain barrier (BBB). Tightly connected brain microvascular endothelial cells (BMVECs) of the barrier limit passage of most molecules. However, circulating macrophages known to traverse the BBB are frequently found in GBMs both in animal studies and patient biopsies.^14^ Using an in vitro model of the BBB, we successfully demonstrated delivery of GNPs loaded in macrophages to GBM9+ tumour cells located across an intact barrier. Given the dismal prognosis of GBM9+ tumours, our study paves the way for  a new and combinatorial strategy that is much needed to target this subset of GBM.

## 2 MATERIALS AND METHODS

### 2.1 Cell culture and plasmids

Human brain microvascular endothelial cells (hBMVECs) were maintained in M199 media (Invitrogen) supplemented with 10% foetal bovine serum (Sigma) and 5% antibiotic‐antimycotic (10 000 U/mL penicillin, 10 000 mg/mL streptomycin, Gibco). U251n and U87 were cultured in DMEM (Gibco) and supplemented with 10% foetal bovine serum (Sigma) and 5% antibiotic‐antimycotic (Gibco). RAW264.7 cells were cultured in DMEM (Gibco) and supplemented with 10% foetal bovine serum (Sigma). All cells were maintained in a 5% CO_2_ incubator at 37°C. Full‐length wild‐type mNHE9‐EGFP and mNHE9‐mcherry and S438P mutant were cloned into FuGW lentiviral vector as previously described.^5^ Empty vector (FuGW) was used for control transductions. Viral Core Facility of the University of Michigan executed lentiviral packaging of the virus.

### 2.2 Synthesis of gold nanoparticles with silica cores

Gold nanoparticles with silica cores were made with minor modifications (Appendix S1).^12^ Briefly, changes included increased five wash cycles after silica core synthesis. Refluxing and stirring were carried out for 1.5 hours to allow for increased attachment of TSPEI to SiO_2_. Finally, formic acid was the reducing agent instead of l‐ascorbic acid. The bare and gold nanoshell‐coated particles were characterized by transmission electron microscopy (TEM) and UV‐Vis spectroscopy. The silica particle concentration was calculated based on the average volume of each individual core particle and the total silica volume for a given amount of TEOS (32). GNPs represent gold nanoshell particles with silica cores.

### 2.3 Cellular GNP loading and determination of GNP concentration inside the cells

U251n, U87 and RAW264.7 cells were grown to 85% confluency and then loaded with GNPs at desired concentrations for 4 hours in serum‐free culture media. Cells were then incubated at 37°C for 30 minutes in 10 µmol/L Calcein Blue AM (Thermo Fisher Scientific) and imaged after three quick washes with phosphate buffered saline. Cells not loaded with GNPs served as control. The fluorescent intensity of calcein was quantified as mean fluorescent intensity (MFI) initially by flow cytometry and microscopy. The fluorescence intensity was linearly reduced as the dose of GNPs increased. As the results were very similar by both methods, we used microscopy as the approach for relative quantification. Absolute quantification was conducted using inductively coupled plasma mass spectrometry (ICP‐MS). For ICP‐MS, GNP‐loaded cells were lysed with mammalian cell protein extraction reagent (Thermo Fisher Scientific) that included protease inhibitor mixture (halt protease inhibitor mixture; Thermo Fisher Scientific). Lysates were centrifuged at 13 000 × g for 15 minutes (4°C). The supernatants were dissolved in 2% hydrochloric acid for 2 hours before collecting data (PerkinElmer). The gold concentrations for each lysate sample were normalized to the cellular protein concentration, determined by BCA assay (Thermo Fisher Scientific).

### 2.4 qPCR analysis

mRNA was isolated from U251n and U87 cells using the RNeasy Mini Kit (Qiagen) following manufacturer's instructions with an additional step to remove DNA using DNase I (Ambion, Thermo fisher Scientific). High‐Capacity RNA‐to‐cDNA Kit (Applied Biosystems) was used to synthesize cDNA, following manufacturer's instructions. TaqMan Fast Universal PCR Master Mix (Applied Biosystems) was used to conduct quantitative real‐time PCR analysis, according to the manufacturer's instructions, on CFX connect real‐time system (Bio‐Rad Laboratories). TaqMan gene expression assay probes used were Mm99999915_g1 (GAPDH), Mm00626012_m (NHE9) and Hs03003631_g1 (18s rRNA). Cycle threshold (*C_t_*) values were first normalized to endogenous controls (GAPDH and 18srRNA). Fold change was calculated as 2^−ΔΔ^ *^Ct^*, where ΔΔ*C_t_* is the normalized cycle threshold value relative to control. Three technical replicates of at least three biological replicates were run to account for variance in assays.

### 2.5 Endosomal pH measurement

Endosomal pH measurements were conducted using our previously published protocols.^10^ Briefly, U251n cells plated in fluorodishes (World Precision Instruments) were placed on ice for 10 minutes and then rinsed with cold imaging buffer (Live Cell Imaging Solution (Thermo Fisher Scientific) with 20 mmol/L glucose and 1% BSA) to remove residual serum transferrin. Cells were then incubated with 50 µg/mL pH‐sensitive transferrin (fluorescein‐conjugated transferrin, Tfn‐FITC; Thermo Fisher Scientific), in imaging buffer for 30 minutes. LCIS was used to rinse the cells, following which fluorescence images were acquired (excitation 494 nm and emission 518 nm) with Lumascope 620 (Etaluma). Internal fluorescence was quantified using ImageJ 15 software, and average fluorescence intensity was recorded. NHE9‐mcherry was transfected using Lipofectamine 2000 for expression in U251n cells. Tfn‐FITC fluorescence was quantified only in mcherry‐positive cells. To normalize for total transferrin uptake, pH‐insensitive transferrin (50 µg/mL Alexa Fluor 568‐conjugated transferrin (Tfn‐568) was loaded. A pH calibration buffer kit (Thermo Fisher Scientific) was used to generate a standard curve from which endosomal pH was determined.

### 2.6 Indirect immunofluorescence

U251n cells on coverslips were washed twice with phosphate buffered saline (PBS). The cells were then fixed for 20 minutes at room temperature with solution containing 4% paraformaldehyde and 4% sucrose in PBS, following previously published protocol.^10^ Three washes with PBS were used to remove the fixing solution. Cells were then incubated for a half‐hour in block solution (1% BSA, 0.3 mol/L glycine, and 0.1% Tween 20). For co‐localization experiments with NHE9‐GFP, primary antibodies Rab 5 (Cell Signaling Technology), Rab 11 (Cell Signaling Technology) and LBPA (Echelon) were diluted 1:100 in block solution without Tween 20 and incubated overnight at 4°C. Following PBS washes, Alexa Fluor‐conjugated secondary antibodies (Invitrogen) were used at 1:1000 dilutions for 30 minutes. Cells were mounted onto slides using Prolong gold antifade reagent (Invitrogen). Immunostaining of human brain microvascular endothelial cells (BMVECs) in culture with RAW264.7 cells was conducted as described previously.^10^ Anti‐human von Willebrand factor antibody (DakoCytomation) was used as a marker for BMVECs. All slides were imaged using Lumascope‐620 microscope (Etaluma).

### 2.7 Inhibition of clathrin‐mediated endocytosis

U251n and U87 cells were pre‐incubated in the presence or absence of 25 µmol/L Pitstop‐2 (Sigma) for 25 minutes or 80 μmol/L of dynasore (Sigma) for 30 minutes following previously published protocols 16, 17, 18 before loading with GNP. For transferrin uptake experiments, the cells were serum starved for 30 minutes and then incubated with 75 µg/mL of Alexa Fluor 568‐conjugated transferrin for 15 minutes. For these experiments, Pitstop‐2 was added during the last 10 minutes of serum starvation and continued during the 15 minutes of transferrin incubation.

### 2.8 NEPTT and cell death analysis

Gold nanoparticles‐loaded cells were irradiated in wells of 96‐well plate using a laser (3 W), with beam diameter 2 mm, which was positioned seven inches above the well to illuminate the full area of the well of the 96‐well plate. Two major processes by which NEPTT induces cell death are apoptosis and necrosis. We used Apoptosis and Necrosis Quantitation Kit Plus (Biotium) for quantifying apoptotic and necrotic cells using fluorescence microscopy. An equal number of cells plated on two coverslips were loaded with GNPs. One set of cells was irradiated with 808 nm light and the other set of cells not subjected to irradiation were used as control. Cell death was assayed within an hour after the treatment.

Following manufacturer's instructions, the cells were washed twice with PBS followed by the addition of two staining solutions. Annexin V‐488 stains apoptotic cells green by binding phosphatidylserine (PS) on the cell surface and ethidium homodimer‐III a nucleic acid probe that stains necrotic cells red were the two stains supplied with the kit. After a 15‐minute incubation in dark, the coverslips were washed and cells imaged using Texas red and FITC filters. Fluorescence intensity was quantified using ImageJ software. A second approach we used to analyse apoptosis was Terminal deoxynucleotidyl transferase (TdT) dUTP Nick‐End Labeling assay (TUNEL). This was conducted using In Situ Cell Death Detection (fluorescein) Kit (Roche). The assay was performed according to the manufacturer's protocol. Briefly, an equal number of GNP‐loaded NHE9 overexpressing cells were plated in an 8‐well glass slide (Nunc). While the control group was not irradiated, laser treatment as described previously was given to the experimental group (NEPTT). Cell death was assayed within an hour after the treatment. Slides were washed three times with PBS and embedded in an antifade mounting media containing DAPI. Slides were examined and imaged using a Lumascope‐620 microscope (Etaluma). The number of TUNEL positive cells was counted from discrete areas from each of the three independent experiments, and the average number of TUNEL positive cells was plotted.

### 2.9 Cell proliferation assay

Cells were plated simultaneously in equal numbers and allowed to grow 85% in 96‐well plates. Cells designated to be loaded with GNPs were loaded and immediately returned to the incubator for 6 hours while cells designated not to be loaded with GNPs also remained incubating during this time. For other experiments, an equal number of control and NHE9 overexpressing cells were loaded with GNPs and subjected to treatments as indicated in the figure legends. Cell growth was monitored using the CellTiter 96 Aqueous One Solution Cell Proliferation Assay (MTS, Promega), following manufacturer's instructions. Briefly, 48 hours after the indicated treatments, 20 μL of CellTiter 96 Aqueous One Solution reagent was added to each well of cells in a 96‐well plate and incubated for 2 hours at 37°C. The absorbance was measured at 490 nm using a plate reader.

### 2.10 Macrophage migration assay

The tumour tropic ability of RAW264.7 cells was examined by a transwell migration assay, as described previously.^19^ Briefly, RAW264.7 cells loaded with or without GNPs were suspended in serum‐free culture  media and plated in the upper chamber of the transwell. The lower chamber of the transwell was filled with serum‐free  media in which U251 and U87 cells were cultured for 48 hours (conditioned media). For negative control, the lower chamber was filled with culture media without serum. The RAW264.7 were incubated in the transwell chambers under these conditions for 6 hours, following which the cells remaining in the upper chamber were removed with a cotton swab. The cells that migrated to the other side of the transwell were detached and total protein levels were estimated using BCA assay kit (Thermo Fisher).

### 2.11 In vitro model of blood‐brain barrier and trojan horse delivery of GNPs to glioblastoma cells

Human brain microvascular endothelial cells (hBMVECs) were cultured by seeding growth  media containing 7 × 10^4^ cells in the upper chamber. Transendothelial electrical resistance (TEER) was monitored using EVOM volt‐ohmmeter (World Precision Instruments). The average TEER of the monolayers on day 6 was ~600 Ohm‐cm^−2^ and did not change significantly thereafter. U251 or U87 cells grown to confluence on a coverslip were placed in the lower chamber of the 6‐well plate insert (ThinCert, 0.4 micron transparent insert, USA Scientific) where hBMVECs were cultured for 5 days. 20 ng/mL TNF‐α was also added on day 5 to induce overexpression of cell adhesion molecules in hBMVECs, as described previously.^19^, ^20^ The culture  media in the upper chamber was replaced with GNP‐loaded RAW264.7 cells in culture  media with FBS on day 6 and incubated for 12 hours. The transwells were disposed, and the U251 or U87 cells on coverslips were washed and incubated with Calcein Blue for 30 minutes as described above. The mean fluorescence intensity in U251 and U87 cells was determined by microscopy.

## 3 RESULTS

### 3.1 NHE9 expression elevates GNP uptake in glioblastoma cells

**Figure 1:**
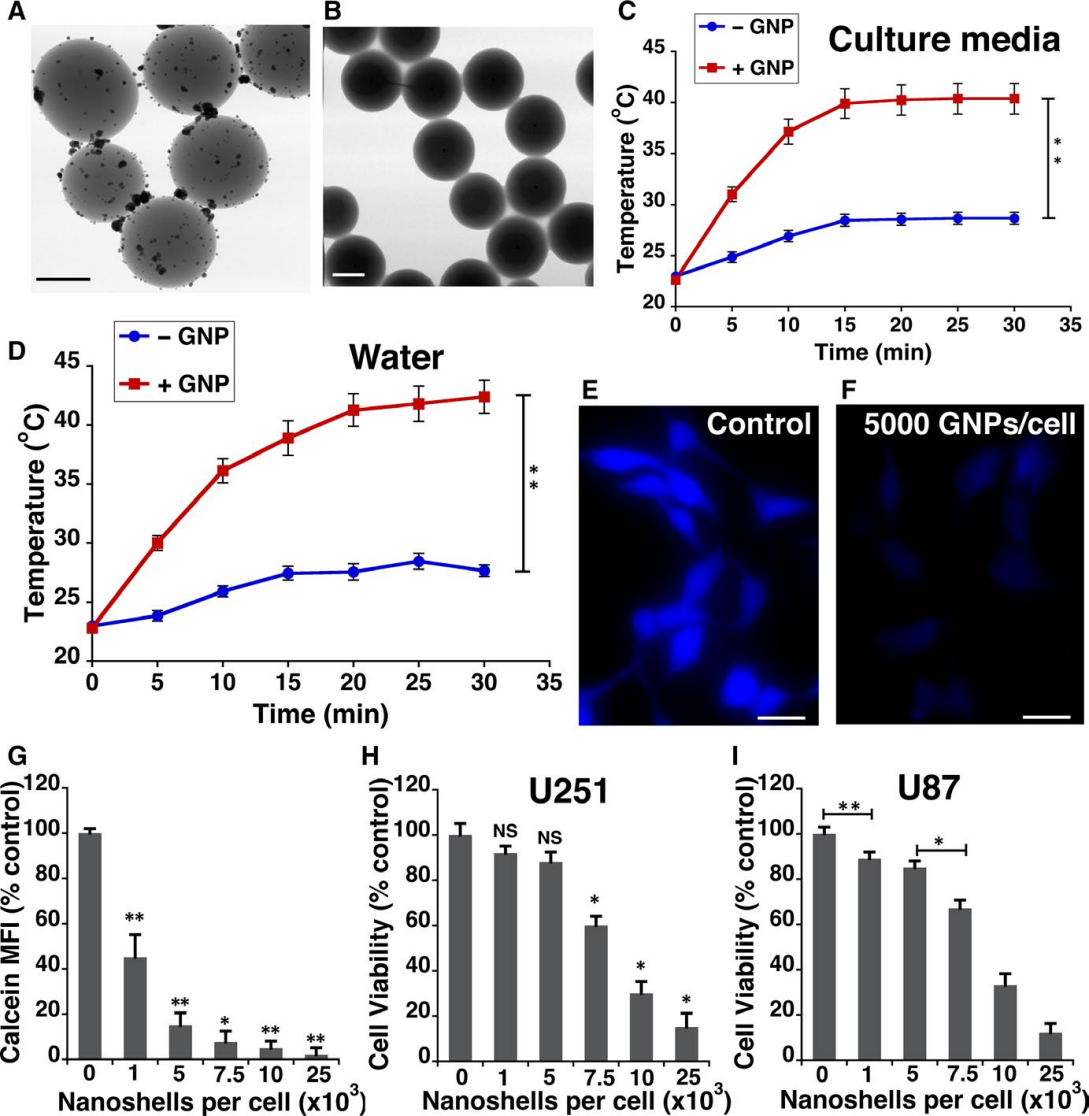
Characterization of gold nanoparticle (GNP) uptake by glioblastoma cells. A, Transmission electron microscopy (TEM) image of synthesized 211 ± 5.5 nm diameter silica core with attached gold nanoparticle seeds, generated by in situ reduction of surface‐bound [AuCl_4_]^−^ ions. Scale bar (black line) is 0.2 µm. B, TEM image of ~271 nm diameter gold nanoshell particle. Scale bar (white line) is 0.2 µm. Comparative time‐dependent heat profiles of cell culture media (C) and water (D), without GNPs (− GNP: blue circle) and with dispersion of GNPs (+ GNP: red squares) under illumination of an 808 nm laser. Representative Calcein Blue fluorescence microscopy images of U251 cells without GNPs (E) and with GNPs (F). Scale bar (white line) is 50 µm. G, U251 Cellular uptake of different concentrations of GNPs, quantified as mean fluorescent intensity (MFI) of Calcein Blue. At least 30 cells were used for quantification at each dosage. Viability of U251 cells (H) and U87 (I) loaded with GNPs was determined by MTS assay, 48 h after loading. Absorbances at 490 nm for each sample were normalized to absorbance of U251 cells not loaded with GNPs. The error bars represent standard deviation (SD) **P < .01 and *P < .05, significant differences relative to control are indicated by the asterisks. NS indicates no significant difference relative to control. Statistical analysis was done using Student's *t* test. Graphs represent an average of at least three biological replicates

Silica cores of 211.15 ± 5.5 nm in diameter were synthesized as described in our previously published.^21^ The surface of the silica core was modified by trimethoxysilylpropyl‐modified polyethyleneimine (TSPEI). This modification provides positively charged imine nitrogen on the surface that enable capture of negatively charged [AuCl_4_]^−^ ions via electrostatic interactions. Reduction of the complex [AuCl_4_]^−^ on the silica core surface, using formic acid, leads to the generation of gold nanoparticle seeds (Figure 1A). These seeds were used as nucleation sites to grow ~59 nm gold shell (Figure 1B and Table S1).^12^ These particles (silica core with gold nanoshell, Figure 1B) are referred to as gold nanoparticles (GNPs) henceforth. GNPs exhibited a broad spectral absorption from the visible to the near‐infrared (NIR) region with a peak centred around 560 nm. GNP‐enabled photothermal therapy is normally induced by irradiation in the NIR region, as absorption by biomolecules such as haemoglobin and melanin is reduced within this optical window.^22^ We assessed the ability of GNPs to convert light energy from a NIR laser (808 nm) to heat by measuring temperature change in both water and cell culture media without serum (Figure 1C‐D). In both water and the culture media within 5 minutes of irradiation, a more than 6°C temperature increase was observed.

It is widely accepted that GNPs enter cells mostly via endocytic pathways. However, the factors that influence cellular uptake of GNPs are still not completely clear. To determine the non‐toxic dosage of GNPs that are in U251 and U87 glioblastoma cells, we used fluorescence quenching of Calcein Blue molecules.^23^ Calcein Blue AM is a cell‐permeant esterase substrate that is weakly fluorescent outside the cell. Once internalized, it accumulates in endosomal compartments where the AM esters are cleaved by esterases to yield highly fluorescent molecules. However, near the surface of GNPs, the fluorescence signals are significantly quenched.^24^ The fluorescence intensity of Calcein Blue linearly reduced with increasing dose of GNPs (Figure 1E‐G). However, doses above 5000 GNPs/cell resulted in a significant loss in viability of U251 cells (Figure 1H) and U87 cells (Figure 1I). Therefore, a dose of 5000 GNPs/cell was used for the remaining experiments.

**Figure 2:**
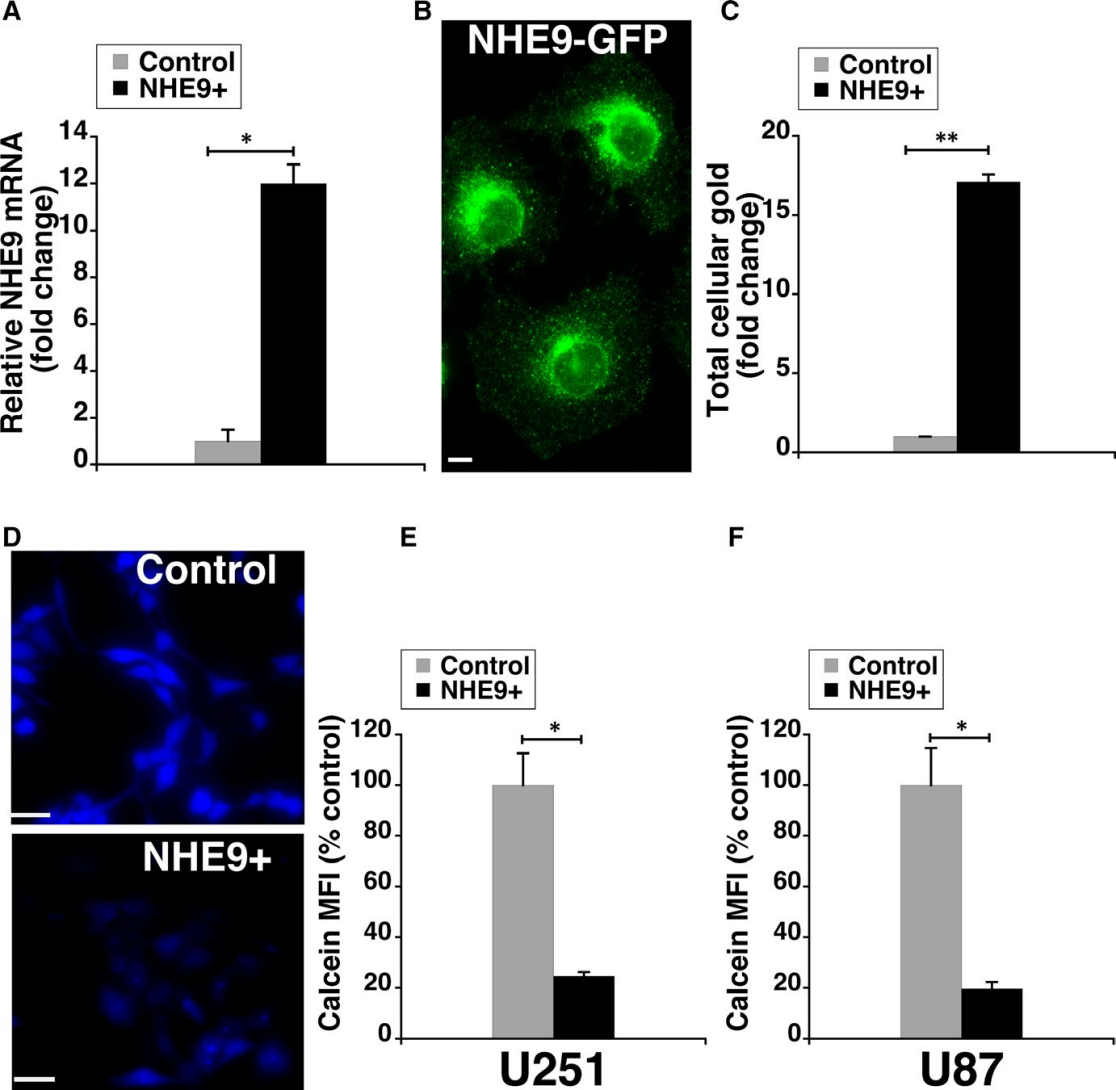
NHE9 expression positively correlates with cellular gold nanoparticle (GNP) accumulation. A, qPCR analysis showing the efficacy of ectopic expression of NHE9‐GFP in U251 cells. The data are plotted as average fold change of mRNA levels relative to control. B, NHE9‐GFP expression in live U251 cells determined by immunofluorescence microscopy 48 h after transduction. Scale bar (white line) is 10 µm (C) Cellular gold concentrations in U251 cells were measured by inductively coupled plasma mass spectrometry (ICP‐MS) and normalized to total cellular protein. The data are plotted as average fold change relative to control. D, Representative Calcein Blue fluorescence microscopy images from GNP‐loaded control (top panel) and NHE9 overexpressing U251 cells (bottom panel). Scale bar (white line) is 50 µm. Cellular GNP levels in control and NHE9 overexpressing U251 (E) and U87 (F) cells, quantified via mean fluorescent intensity (MFI) of Calcein Blue and plotted as per cent control. The error bars represent standard deviation (SD) **P < .01 and *P < .05. Statistical analysis was done using Student's *t* test. Graphs represent an average of at least three biological replicates

Previous studies in glioblastoma cells revealed a general increase in membrane receptors and endocytic uptake upon NHE9 expression.^4^, ^5^, ^10^ Hence, we expected glioblastoma cells transduced with NHE9‐GFP to have significantly higher GNP uptake relative to control glioblastoma cells. Transcript analysis by quantitative PCR revealed an eightfold increase in NHE9 expression in U251 glioblastoma cells (Figure 2A). We also confirmed NHE9‐GFP expression by immunofluorescence in U251 cells (Figure 2B). Next, we compared the cellular uptake of GNPs in U251 cells overexpressing NHE9 with control cells using inductively coupled plasma mass spectrometry (ICP‐MS). We observed ~17‐fold increase in GNP uptake in NHE9 overexpressing cells relative to control (Figure 2C). Consistent with this observation, calcein fluorescence quenching experiments confirmed the increase in GNP uptake with ectopic expression of NHE9 in U251 (Figure 2D‐E) and U87 cells (Figure 2F). We did not observe any significant difference in Calcein Blue fluorescence between control and glioblastoma cells overexpressing NHE9, in the absence of GNPs. These results clearly indicate that increased NHE9 expression correlates positively with GNP accumulation in glioblastoma cells.

### 3.2 Elevation in uptake of GNPs occurs via clathrin‐mediated endocytosis

**Figure 3:**
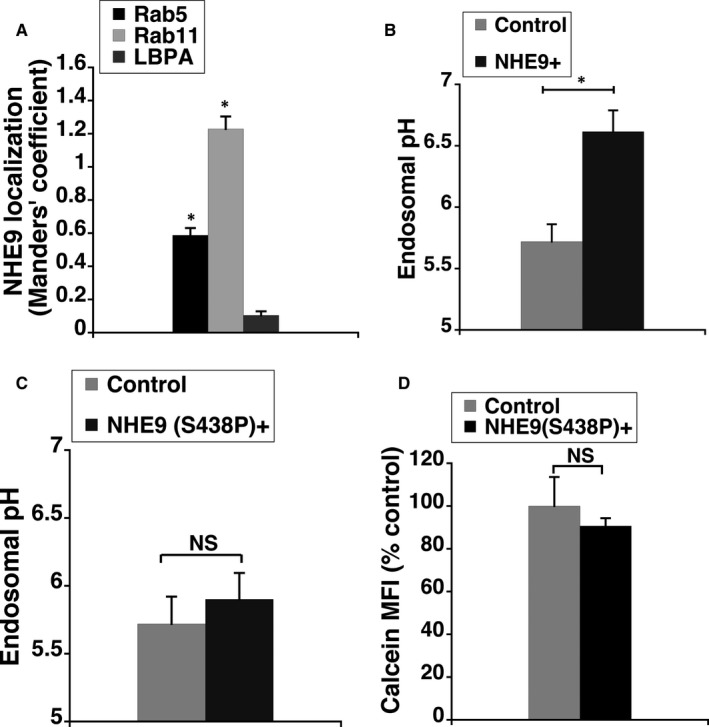
Endosomal pH modulation by NHE9 effects gold nanoparticle (GNP) uptake. A, Subcellular localization of NHE9‐GFP with early (Rab5), recycling (Rab11) and late endosome (LBPA) markers was determined by immunofluorescence microscopy, in U251 cells. Quantification of NHE9‐GFP localization with the indicated organellar markers is shown using Manders’ coefficient. At least 30 cells were used for quantification for each marker. B, pH in endosomal compartments is alkalinized in U251 cells overexpressing NHE9 relative to control. Linear calibration curve of endosomal pH from fluorescence ratio of internalized transferrin tagged to pH‐sensitive and pH‐insensitive probes was generated and endosomal pH was measured as described in the methods section. C, Expression of the previously characterized autism‐associated NHE9 functional mutant (S438P) did not result in significant alkalization of endosomal pH. D, Expression of S438P mutant of NHE9 did not result in significant increase in GNP uptake by U251 cells. GNP uptake was quantified via mean fluorescent intensity (MFI) of calcein and plotted as per cent control. The error bars represent SD *P < .05 and NS = not significant. Statistical analysis was done using Student's *t* test. Graphs represent an average of at least three biological replicates

To determine the mechanism for elevated uptake of GNPs by glioblastoma cells overexpressing NHE9, the subcellular localization of NHE9‐GFP in U251 cells was examined. Similar to our previous observations in normal glia and U87 cells,^5^, ^8^ NHE9 localized predominantly to early and recycling endosomes in U251 glioblastoma cells (Figure 3A). Consistent with its role in transporting protons out of the endosome in exchange for sodium or potassium ions, overexpression of NHE9 resulted in alkalizing the endosomal lumen by ~1 pH unit (Figure 3B). To determine whether the increased GNP uptake was a result of NHE9’s ion exchange activity and not some non‐specific effect of NHE9 overexpression, we evaluated the effect of a previously described loss‐of‐function mutation (S438P) in NHE9, on GNP uptake.^5^ This mutant protein failed to significantly effect endosomal pH (Figure 3C), despite localizing to sorting and recycling endosomes similar to the wild‐type NHE9 (data not shown). Concurrently, no increase in GNP uptake was observed (Figure 3D). This confirmed the increase in GNP uptake is due to NHE9 overexpression.

**Figure 4:**
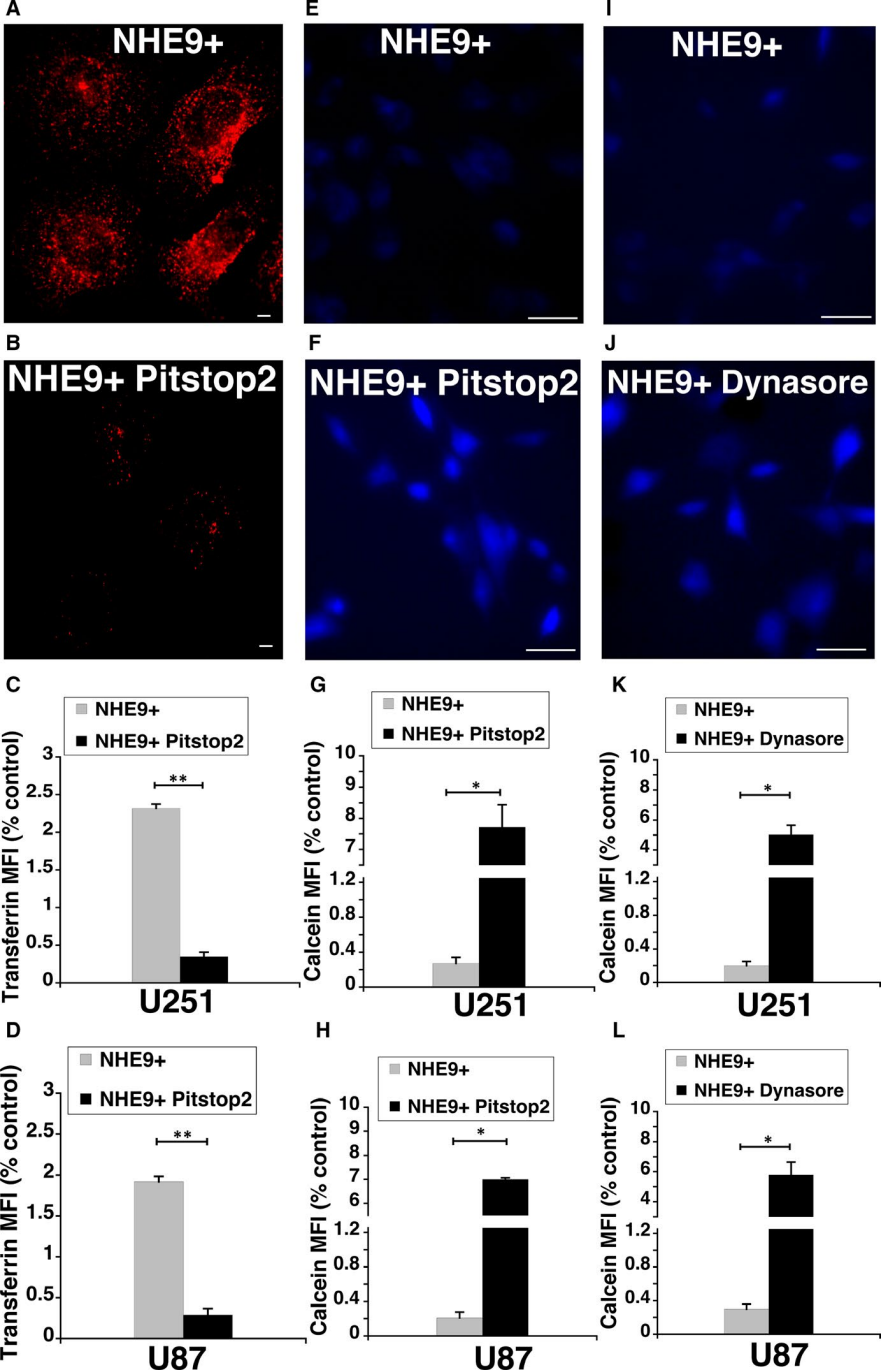
NHE9 overexpression elevates gold nanoparticle (GNP) uptake via clathrin‐mediated endocytosis. Representative fluorescence micrographs showing accumulation of Alexa Fluor‐tagged transferrin (Tfn‐568) in NHE9 overexpressing U251 cells that were untreated (A) or treated with Pitstop2 (B). Scale bars (white lines) are 10 μm. Plot represents mean fluorescence intensity of Tfn‐568 calculated from fluorescence micrographs of U251 (C) and U87 cells (D). Treatment of U251 cells overexpressing NHE9 with Pitstop2 results in decreased transferrin uptake. Representative fluorescence micrographs showing accumulation of Calcein Blue in NHE9 overexpressing U251 cells that were untreated (E) or treated with Pitstop2 (F). Scale bars (white lines) are 50 μm. Plot represents mean fluorescence intensity of Calcein Blue calculated from fluorescence micrographs of U251 (G) and U87 cells (H). Representative fluorescence micrographs showing accumulation of Calcein Blue in NHE9 overexpressing U251 cells that were untreated (I) or treated with dynasore (J). Scale bars (white lines) are 50 μm. Plot represents mean fluorescence intensity of Calcein Blue calculated from fluorescence micrographs of U251 (K) and U87 cells (L). At least 30 cells were used for fluorescence quantification in each experiment. Treatment of U251 and U87 cells overexpressing NHE9 with Pitstop2 and Dynasore results in decreased GNP uptake based on inverse relation with mean fluorescence intensity of Calcein Blue. The error bars represent standard deviation (SD) **P < .01 and *P < .05. Statistical analysis was done using Student's *t* test. Graphs represent an average of at least three biological replicates

Clathrin‐mediated endocytosis (CME) facilitates the internalization and recycling of receptors engaged in transporting various cargos including GNPs. We hypothesized that the increase in GNP uptake due to NHE9 overexpression is mediated through CME. To test this hypothesis, we evaluated the effect of CME inhibitor Pitstop2 on the uptake of GNPs in NHE9 overexpressing U251 and U87 glioblastoma cells. Coated pit assembly and dissociation required for internalization of plasma membrane receptors is organized by clathrin via its terminal domain. Pitstop2 has been shown to selectively block endocytic ligand association with the clathrin terminal domain.^17^ Incubation for 25 minutes with Pitstop2 resulted in a ~10‐fold inhibition of transferrin uptake, a known cargo for transport via CME (Figure 4A‐D). Similarly, Calcein Blue fluorescence in NHE9 overexpressing cells treated with Pitstop2 was ~30‐fold higher relative to control, indicating a drastic decrease in GNP uptake (Figure 4E‐H). To confirm the decrease in GNP uptake was specifically due to CME inhibition, we examined the effect of another CME inhibitor, dynasore.^18^, ^25^, ^26^ Treatment with dynasore, a cell‐permeable inhibitor of dynamin GTPases that disrupts CME, also resulted in a significant increase in Calcein Blue fluorescence relative to untreated cells (Figure 4I‐L). Together, these observations support the idea that alkalization of endosomal lumen by NHE9 leads to elevated clathrin‐mediated endocytic uptake of GNPs.

### 3.3 NEPTT induces cell death via apoptosis

**Figure 5:**
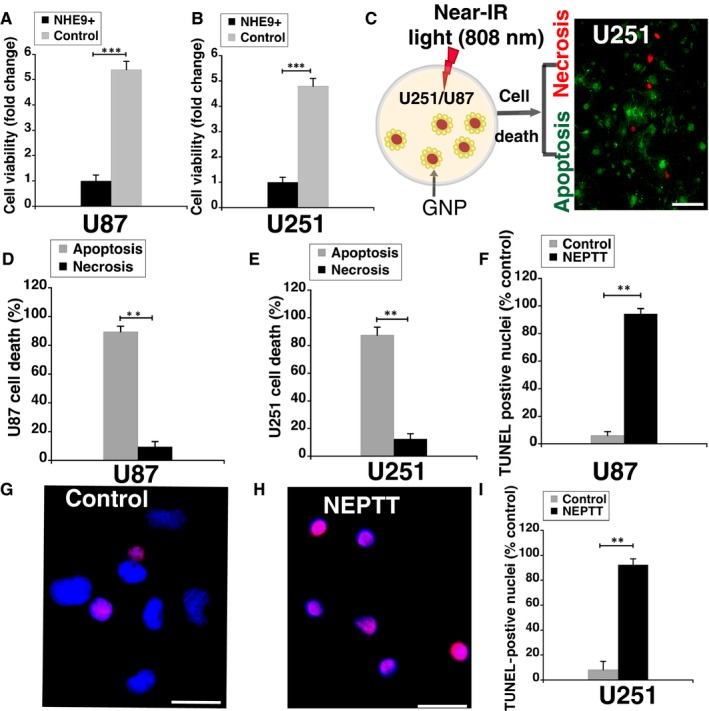
Apoptosis is the predominant mode of cell death induced by NEPTT of U251 cells overexpressing NHE9. Normalized cell proliferation as determined by MTS assay of U87 (A), and U251 (B) control and NHE9 overexpressing cells subjected to NEPTT. C, Schematic representation of the experiment showing gold nanoparticles (GNPs)‐loaded GBM cells subjected to irradiation with 808 nm laser. Apoptosis and necrosis are the two mechanisms of cell death induced by NEPTT. While necrosis may lead to pro‐inflammatory responses that could negatively impact treatment success, apoptosis is the preferred mechanism as it does not elicit such responses. Representative micrograph of dual staining of U251 cells with Annexin V (green, apoptosis) and ethidium homodimer‐III (red, necrosis) after NEPTT is shown. Scale bar (white line) is 100 µm. Quantitative analysis of apoptotic and necrotic cell death in U87 (D) and U251 (E) cells subjected to NEPTT. Annexin V labelling was used to stain apoptotic cells and ethidium homodimer‐III was used to stain necrotic cells. The plot shows quantification from fluorescence microscopy. At least 30 cells were used for quantification. F, Quantitative analysis of apoptotic cell death in U87 cells by TUNEL assay, from fluorescence micrographs. Cells loaded with GNPs were either irradiated with 808 nm laser (NEPTT) or not subjected to irradiation (Control). At least 30 cells were used for quantification. G, H, Representative merged images showing apoptotic TUNEL positive (red) and healthy DAPI‐stained (blue) U251 cells overexpressing NHE9. I, Quantitative analysis of apoptotic cell death in U251 cells by TUNEL assay, from fluorescence micrographs. Scale bar (white line) is 50 µm. The error bars represent SD ***P < .001 and **P < .01. Statistical analysis was done using Student's *t* test. Graphs represent an average of at least three biological replicates

NEPTT‐enabled ablation of cancer cells depends on efficient thermal conversion of near‐infrared light by GNPs. Consistent with increased GNP uptake leading to higher temperatures within the treatment time, a ~4‐fold and ~5‐fold lower cell viability was observed in U251 and U87 glioblastoma cells overexpressing NHE9, respectively, relative to control glioblastoma cells (Figure 5A‐B). With GNP alone or laser irradiation alone, no significant differences in cell viability between NHE9 overexpressing and control glioblastoma cells were observed (data not shown). However, photothermal therapy is known to induce cell death by apoptosis and necrosis depending on irradiation parameters.^27^

Apoptosis is the preferred way to induce cell death as primary necrosis leads to pro‐inflammatory responses. To evaluate the type of cell death induced by near‐infrared irradiation (808 nm), we conducted dual staining of cells with Annexin V (stains apoptotic cells) and ethidium homodimer‐III (stains necrotic cells; Figure 5C). Quantitative comparison of the staining showed that apoptotic cell death was significantly higher than necrotic death in both U251 and U87 cells (Figure 5D‐E). As a complementary approach, TUNEL staining was used to label fragmented DNA generated during apoptosis. Greater than 90% of the cells were TUNEL positive in GNP‐loaded U87 (Figure 5F) and U251 cells (Figure 5G‐I) overexpressing NHE9, after irradiation. Together, this evidence confirms that NEPTT initiates apoptosis to induce cell death, which is higher in glioblastoma cells overexpressing NHE9.

### 3.4 Macrophages mediate delivery of GNPs to glioblastoma cells across an intact blood‐brain barrier

**Figure 6:**
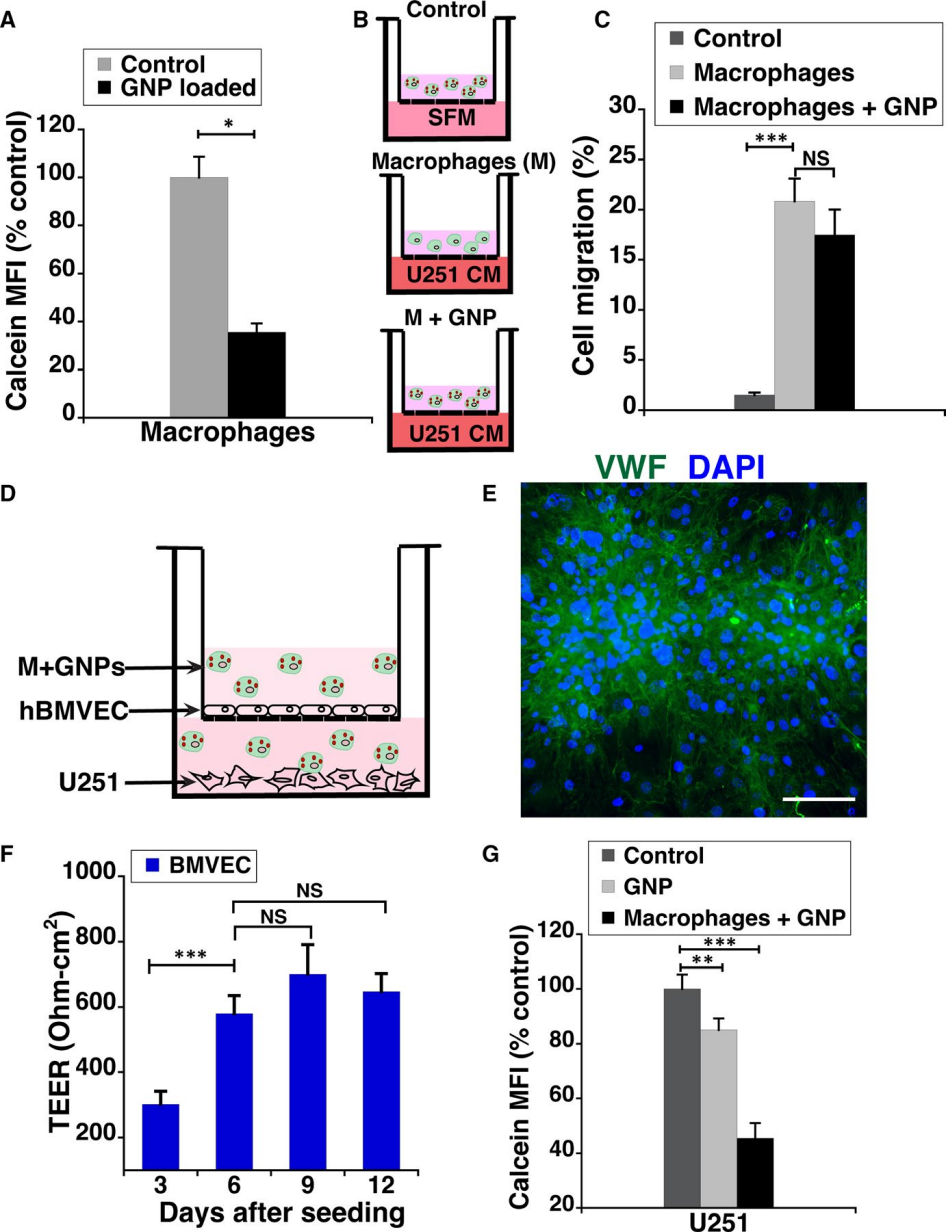
Macrophages as carriers boost the transcytosis of gold nanoparticles (GNPs) across the endothelial barrier and delivery to U251 cells. A, Uptake of GNPs by RAW264.7 cells (macrophages), quantified as mean fluorescent intensity (MFI) of Calcein Blue and plotted as per cent control. B, Schematic illustration of experimental set‐up for demonstration of RAW264.7 cell chemotaxis. In the negative control, GNP‐loaded macrophages were added to the top chamber and serum‐free medium (SFM) was added to the bottom chamber. For the experimental conditions, macrophages without GNPs or loaded with GNPs were added to the top chamber and U251 conditioned media (CM) was added to the bottom chamber. After incubation as described in methods, cells remaining on the upper membrane of the transwell were removed with a cotton swab and cells migrated to the lower membrane surface were detached and protein amount quantified using BCA assay. C, Plot showing quantitative comparison of macrophage chemotaxis based on the protein amount detected by the BCA assay. D, Schematic illustration of in vitro endothelial barrier model for macrophage‐mediated GNP delivery to U251 cells. E, Representative micrograph showing RAW264.7 cells in culture with human brain microvascular endothelial cells (BMVEC) on the transwell filter. Von Willebrand factor (VFW, green) is a marker for endothelial cells. DAPI staining (blue) of nuclei for both cell types is also shown. Scale bar (white line) is 50 µm. F, Transendothelial electrical resistance (TEER) values for BMVECs grown in the transwell model system for 12 days. G, Cellular GNP levels of NHE9 overexpressing U251 cells in the bottom chamber of the transwell, quantified via mean fluorescent intensity (MFI) of Calcein Blue. GNP was added independently or via loaded in macrophages to the upper chamber. No GNPs were added in the upper chamber for the control. The error bars represent SD ***P < .001, **P < .01 and *P < .05. Statistical analysis was done using Student's *t* test. Graphs represent an average of at least three biological replicates

During the early stages of glioblastoma development, the blood‐brain barrier (BBB) is intact and prevents direct delivery of GNPs to tumour cells via blood circulation. It's only later when the integrity of the BBB is compromised by the tumour, the GNPs could potentially be taken up from the blood. To assess the feasibility of delivering GNPs to glioblastoma cells across an intact barrier, we first evaluated the tumour homing ability of RAW264.7 macrophage loaded GNPs.^19^, ^28^ RAW cells were able to contain ~5000 GNPs per cell (Figure 6A), without significant loss in their viability. The migration of free and GNP‐loaded macrophages towards serum‐free DMEM (control) was negligible (Figure 6B). In contrast, ~20% of the GNP‐loaded macrophages migrated to the U251 conditioned media (Figure 6C). A statistically significant difference in the migration ability between free and GNP‐loaded macrophages with U251 conditioned media was not observed (Figure 6C).

With the goal of assessing the transcytosis efficiency of GNP‐loaded macrophages, RAW264.7 cells were used in conjunction with an in vitro model of BBB (Figure 6D‐E). Our experimental design consisted of human brain microvascular endothelial cells (hBMVECs) grown as a monolayer on a transwell membrane, with U251 cells plated in the bottom of the transwell chamber. The integrity of the barrier was assessed by measuring the transendothelial electrical resistance (TEER). The TEER plateaued at ~600 Ωcm^2^ in about 6 days after plating (Figure 6E). To better simulate the tumour microenvironment, 20 ng/mL of TNF‐α known to induce overexpression of cell adhesion molecules in endothelial cells of the BBB was added.^20^ The transcytosis ability of GNPs alone and loaded in macrophages was compared by monitoring the loss in Calcein Blue fluorescence of U251 cells. As shown in Figure 6F, macrophage‐mediated delivery of GNPs is twofold higher than GNP treatment alone. These results clearly indicate that macrophages mediated delivery of our GNPs.

### 3.5 Combinatorial treatment with NEPTT increases the efficacy of EGFR inhibitor

**Figure 7:**
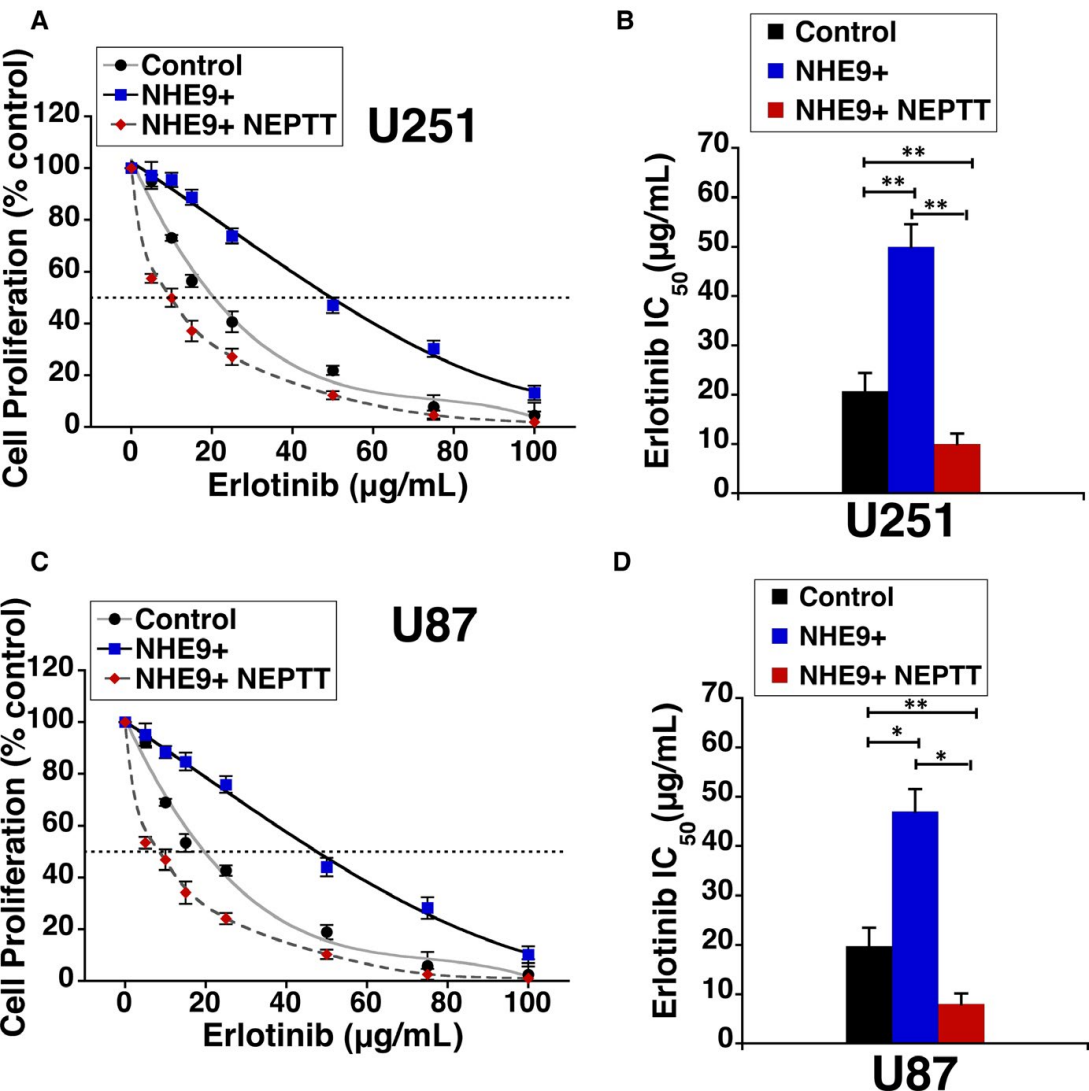
NEEPT attenuates the resistance to erlotinib in NHE9 overexpressing GBM cells. A, U251 control cells, NHE9 overexpressing U251 cells and NHE9 overexpressing U251 cells subjected to NEPTT were treated with different concentrations of EGFR inhibitor erlotinib and cell viability was measured by MTS assay. The dotted line indicates the erlotinib concentration (x‐axis) at which only 50% of cells are viable (y‐axis). B, The graph shows IC_50_ for erlotinib is elevated in cells overexpressing NHE9 relative to control. NEPTT significantly mitigates this resistance. C, Same as (A) with U87 cells. D, Same as (B) with U87 cells. The error bars represent SD **P < .01 and *P < .05. Statistical analysis was done using Student's *t* test. Graphs represent an average of at least three biological replicates

To further explore the therapeutic potential of NEPTT, we evaluated if a combinatorial approach with erlotinib would more effectively inhibit proliferation of tumour cells overexpressing NHE9. Previous studies have shown that NHE9 overexpression exerts post‐translational control by increasing persistence of EGFRs on glioblastoma cell membranes, by attenuating receptor turnover significantly.^4^ Consistent with increased membrane persistence of EGFRs, efficacy of erlotinib, an FDA‐approved receptor tyrosine kinase inhibitor of EGFR, is significantly reduced. The IC_50_ of erlotinib increased from ~20 µg/mL in control cells to ~50 µg/mL in U251 cells overexpressing NHE9 (Figure 7A). The IC_50_ of erlotinib decreased five fold when used in combination with NEPTT (Figure 7B). Results from similar experiments in U87 cells were consistent with what results in U251 cells (Figure 7C‐D). Our data clearly demonstrate a significant increase in efficacy of erlotinib when used in combination with NEPTT.

## 4 DISCUSSION

The pathophysiological mechanism underlying the subset of glioblastomas with NHE9 overexpression (GBM9+) was recently identified.^4^ This provided insight into a hitherto unknown link between endosomal pH and gliomagenesis. NHE9 is a master regulator of cargo delivery and recycling by mediating inside‐out control of oncogenic signalling in glioblastoma.^4^, ^8^ Increased cell surface receptor densities are the hallmark of GBM9+ tumours.^4^, ^8^ This gain of function observed in GBM9+ tumours formed the basis for our study directed at identifying an effective therapeutic strategy for GBM9+ subset of tumours.

**Figure 8:**
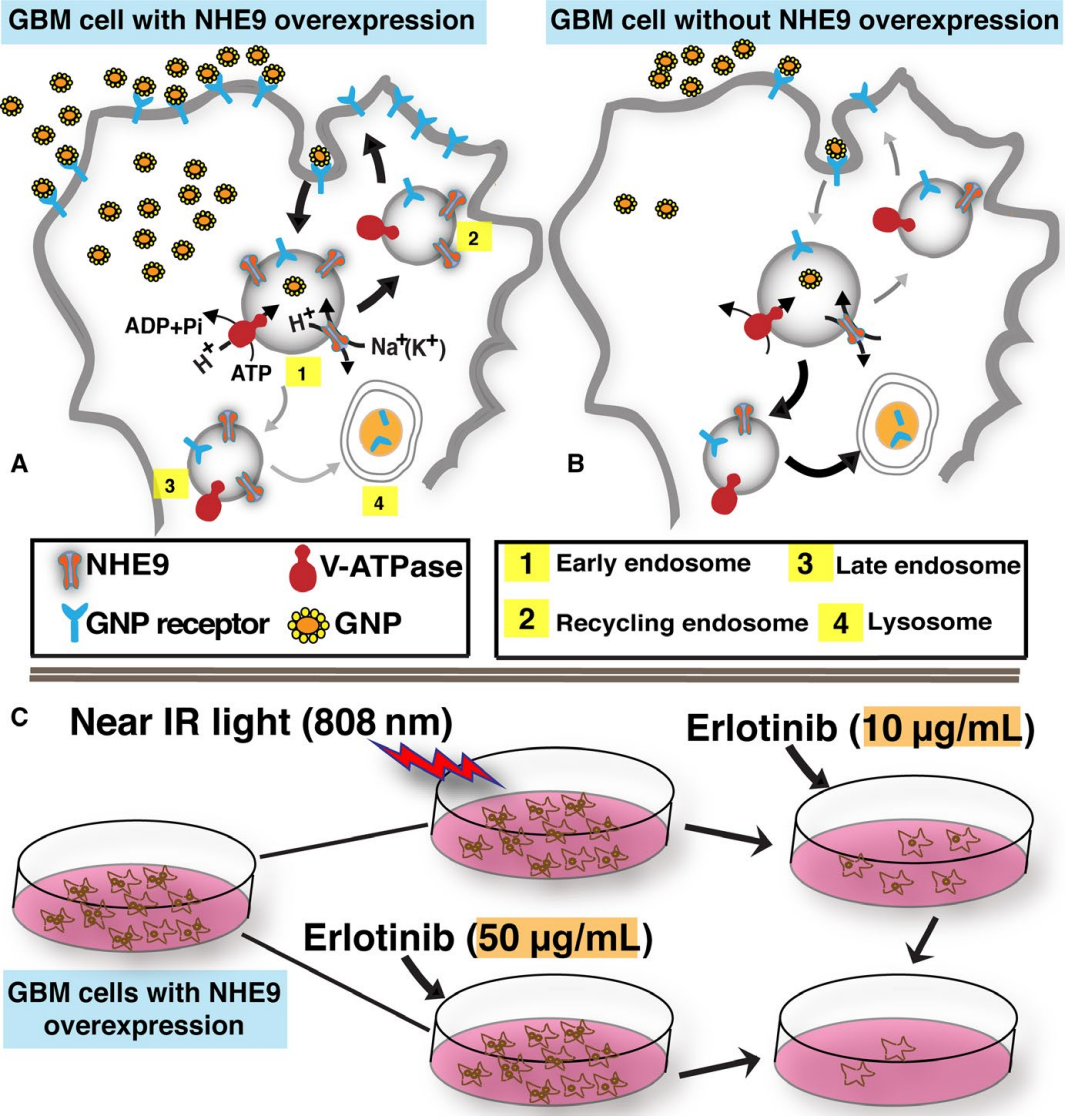
Schematic illustrating NHE9 overexpression in glioblastoma (GBM) enhances NEPTT. NHE9 is localized predominantly to early and recycling endosomes in GBM cells. Overexpression of NHE9 results in alkalizing the endosomal lumen to affect clathrin‐mediated endocytosis (CME). CME facilitates the internalization and recycling of receptors. Endosomal alkalization resulting from NHE9 overexpression has also been shown to divert trafficking of receptors away from lysosome. A combination of increased internalization and recycling with decreased lysosomal degradation potentially leads to increased plasma membrane receptor density, which include receptors that can transport gold nanoparticles (GNPs) into the GBM cells and epidermal growth factor receptors (EGFRs). Increase in EGFRs significantly lowers the efficacy of drugs such as erlotinib that target EGFRs, making NHE9 overexpressing GBM cells very difficult to treat. However, elevation in the number of receptors that transport GNPs can be exploited to accumulate substantially higher number of GNPs in NHE9 overexpressing GBM cells (A), making them more susceptible to NEPTT, relative to cells without NHE9 overexpression (B). Combinatorial treatment with NEPTT increases the efficacy of erlotinib by several fold (C) and thus holds great promise for new therapy targeting NHE9 overexpressing GBM

Gold nanoparticle uptake by mammalian cells is mediated by caveolae‐mediated endocytosis, clathrin‐mediated endocytosis and micropinocytosis.^29^ The specific mode utilized for uptake, however, depends on GNP properties such as surface chemistry, net charge, size, shape and protein corona (protein layer on nanoparticles due to proteins adsorbed from biological fluids).^30^ The GNP uptake parts of our experiments were conducted in serum‐free media, to avoid the effects of serum proteins and alterations to the net charge on GNPs. We observed a ~17‐fold increase in GNP uptake via clathrin‐mediated endocytosis, resulting from a ~10‐fold change in NHE9 transcript levels (Figure 8A‐B). Exceptionally high transport rates of ~1500 ions per second have been estimated for sodium‐proton exchangers.^31^ Therefore, even small perturbations in NHE9 expression could result in big changes in the ionic milieu within the endosomal lumen. However, the specific molecular mechanism(s) linking ion exchange activity of NHE9 to endocytic trafficking is not completely clear. Recently published work by the Brett group suggests that the NHE9 yeast ortholog is important for fusion of the endosomes with lysosomes.^32^ The molecular mechanisms facilitating this are evolutionarily conserved. Changes in NHE9 expression likely affect pH‐sensitive machinery involved in SNARE‐mediated merging of the lipid bilayers on endosomes and lysosomes. This fusion is the last step of endocytosis, required for surface protein degradation.

Modes of nanoparticle delivery for malignant glioblastomas have received much attention during the last few years. In vivo, intravenous injection of GNPs has better therapeutic benefit than direct infusion of GNPs into the tumour.^33^ GNPs accumulate in tumours via enhanced permeability and retention effect by passing through compromised endothelial tumour vasculature, especially during the later stages of the cancer.^34^ GNPs directly infused into the tumour diffuse in the interstitial space around the tumour cells without any tumour cell‐specific localization.^33^ Thus, when GNPs are presented to glioblastoma cells via an intravenous injection, GBM9+ tumour cells could potentially outcompete surrounding normal cells due to both increased endocytic uptake and enhanced permeability and retention. This could have potentially far‐reaching effects not only in terms of treatment effectiveness but also protecting cells from hyperthermia. However, during early stages of glioblastoma, the BBB is still intact impeding the GNPs from entering the tumour. Even in later stages, increased interstitial fluid pressure inside the tumour and the blood tumour barriers need to be overcome for the GNPs to efficiently enter the tumour cells. This report looked at the feasibility of delivering the GNPs we synthesized, to GBM9+ tumour cells across the BBB. Cell‐based carriers have been exploited in recent years as Trojan horses to carry GNPs through barriers into brain tumour sites.^19^, ^35^ Macrophages are a class of white blood cells that can naturally infiltrate most solid tumours including glioblastomas.^19^, ^36^, ^37^ On the endothelial cells of the BBB, cell adhesion molecules (CAMs) overexpressed as result of the tumour inflammation mediate interaction between macrophages and the endothelial cells to facilitate migration across the barrier.^20^ We demonstrate migration of GNP‐loaded macrophages and delivery to GBM9+ tumour cells across the BBB using a transwell monolayer model. Direct contact between macrophages and GBM9+ tumour cells facilitates GNP delivery. In vivo, tumour inflammation environment could facilitate delivery via exocytosis.^19^

In conclusion, while NHE9 overexpression enables the oncogenic potential of a subset of GBMs, it also provides an opportunity for novel precision therapy. NEPTT enhances the efficacy of erlotinib by several fold (Figure 8C). An important focus of our ongoing research is to selectively obliterate cancer cells by optimizing GNP shell thickness, spatiotemporal manipulation of photothermal treatment and encapsulation of erlotinib in the nanoparticles. Encapsulation efficiently reduces drug‐induced toxicity to other cells and could also improve the amount of drug reaching the tumour cells. Future studies will focus on evaluating these findings in nude mice with intracranial GBM9+ tumours.

## CONFLICT OF INTEREST

The authors declare that they have no conflicts of interest with the contents of this article.

## AUTHOR CONTRIBUTIONS

KCK and K.B designed the research. AEP, D.G, L.J and KCK conducted the research. KCK, AEP and K.B analysed the data. KCK wrote the paper. All authors reviewed the results and approved the final version of the manuscript.

## Data Availability

The data that support the findings of this study are available from the corresponding author upon reasonable request.
