# Supplementary material for: A gain of function paradox: Targeted therapy for glioblastoma associated with abnormal NHE9 expression
Source: J Cell Mol Med. 2019 Sep 18;23(11):7859–72. doi: 10.1111/jcmm.14665 (PMC6815843; doi:10.1111/jcmm.14665)
Supplement: Supplementary file 1 [file JCMM-23-7859-s001.docx]

**Supplementary Information**

**Table S1.** Zeta potential values during various steps during synthesis of gold nanoparticles with silica cores

| **Step in Synthesis** | **Zeta Potential (mV)** |
| --- | --- |
| SiO_2_ | -75.10 ± 1.73 |
| Core with TMSSP monolayer | 52.27 ± 0.91 |
| Reduced gold seeds | 36.62 ± 2.45 |
| Gold nanoshells | -14.45 ± 2.18 |

**Supplementary Methods**

**Synthesis of nanoshells with gold seeds on silica cores**

*Formation of silica core nanoparticles*

Silica core nanoparticles were synthesized by adding 75.0 mL of absolute ethanol (200 proof) to 25.0 mL of Milli-Q water and 3.1 mL of 14.5 M NH_4_OH. The solution was stirred at room temperature for 5 minutes, followed by the addition of 3.0 mL of tetraethyl orthosilicate (TEOS). After stirring for 8 hours, the white precipitate was centrifuged at 5000 rpm for 25 minutes, the supernatant liquid was removed, absolute ethanol was added to a final volume of 30.0 mL, and the solution was sonicated for 5 minutes. This washing process was repeated 4 more times.

*Formation of the TSPEI monolayer*

30.0 mL of the silica core solution was dispersed in 60.0 mL of absolute ethanol. While stirring, 10.0 mL of TSPEI (50% in isopropanol) was added. After refluxing for 90 minutes with stirring, the solution was stirred for an additional 90 minutes without refluxing. The solution was then left to sit at room temperature. On the following day, the solution was centrifuged at 5000 rpm for 25 minutes, the supernatant liquid was removed, 95% ethanol was added to a final volume of 30.0 mL, and the functionalized silica nanoparticle cores were sonicated for 5 minutes. This washing process was repeated 4 more times. The pellet was then left to sit overnight.

*Adsorption of ions to TSPEI-functionalized Sicore*

After removing the supernatant liquid, the silica/TSPEI nanoparticle pellet was redispersed in 30.0 mL of a 1 x 10^-2^ M aqueous HAuCl4 solution and thoroughly mixed for 5 minutes. To dark-age the solution, the centrifuge tube was wrapped in aluminum foil and allowed to sit overnight. The solution was then centrifuged at 5000 rpm for 25 minutes, the supernatant liquid was removed, Milli-Q water was added to a final volume of 30.0 mL, and the solution was sonicated for 5 minutes. This washing process was repeated 4 more times.

*In situ generation of gold seeds with formic acid as reducing agent on functionalized silica cores*

A 2% (w/v) aqueous formic acid solution was made by adding 681.8 μL of formic acid to 30.0 mL of water. The solution was stirred for 5 minutes and then added to the silica/TSPEI/ pellet. This solution was thoroughly mixed for 5 minutes then dark-aged in aluminum foil overnight. The solution was then centrifuged at 5000 rpm for 25 minutes, the supernatant liquid was removed, and Milli-Q water was added to a final volume of 30.0 mL (no sonication). This washing process was repeated 4 more times.

*Synthesis of gold nanoshells*

A 25 mM HAuCl_4_ solution was made by dissolving 0.0594 g of HAuCl_4_ in 6.99 mL of water, which was then wrapped in aluminum foil and placed in a refrigerator overnight. The growth solution was then made by adding 0.0143 g of K_2_CO_3_, 0.750 mL of HAuCl_4_, and 50.0 mL of water. This solution was stirred until it became clear, wrapped in aluminum foil, and then placed in the refrigerator to sit overnight. The next day, while stirring, 9 mL of growth solution was added to 100 μL of the silica/TSPEI/Au seeded nanoparticle solution while stirring. After 5 minutes, 80 microliters of formaldehyde was added to both beakers. After stirring for 90 minutes, the solutions sat at room temperature overnight. The solutions were then centrifuged at 5000 rpm for 25 minutes, the supernatant liquid was removed, and Milli-Q water was added to a final volume of 10.0 mL. This washing process was completed 5 times for each.
